# Internet addiction, sleeping habits and psychological distress in Brazilian adolescents and young adults

**DOI:** 10.1186/s41155-024-00323-0

**Published:** 2024-09-14

**Authors:** Maísa Gelain Marin, Antônio Bonfada Collares Machado, Guilherme da Silva Freitas, Rosa Maria Martins de Almeida

**Affiliations:** Programa de Pós-Graduação Em Psicologia, Universidade Federal Do Rio Grande Do Sul (UFRGS), Ramiro Barcelos Street, 2600, Room 216, Bairro Floresta, Porto Alegre, RS 90035-003 Brazil

**Keywords:** Internet addiction, Sleep, Depression, Anxiety, Stress

## Abstract

**Background:**

The internet is widespread in modern society and has raised concerns about excessive usage and its consequences for different aging groups.

**Objective:**

This study explores the relationships between internet addiction, sleep patterns, and psychological distress in adolescents and young adults.

**Materials and methods:**

In order to assess this phenomenon, an exploratory cross-sectional study was conducted in southern Brazil from October to December 2023. A questionnaire, administered via Google Forms, collected data from 618 participants aged 15–36. The participants consisted of a non-probabilistic community sample selected based on convenience criteria. Instruments included a sociodemographic questionnaire, the Internet Addiction Test, Depression, Anxiety, and Stress Scale, and Social Media Engagement Questionnaire.

**Results:**

It was categorized 48.1% as having moderate internet addiction, and classified 0.8% as having severe internet addiction. Correlation analysis revealed positive associations between internet addiction and depression, anxiety, and stress. Logistic regression identified internet addiction and overall psychological distress as predictors of sleep difficulties, along with unemployment status. The findings highlight the detrimental impact of excessive internet use on sleep quality and mental health. The bidirectional relationship between internet addiction and psychological distress underscores the complex interplay between these variables.

**Conclusions:**

This study underscores the urgent need for interventions targeting internet addiction and its associated consequences in adolescents and young adults. Addressing internet addiction levels and promoting healthy internet usage habits are crucial steps in mitigating the adverse effects on mental health and sleep quality. There is a need for targeted interventions, protocols, and preventive measures to mitigate the adverse effects of excessive internet use on mental health and sleep quality. Public health strategies should include IA and its consequences in health programs with multidisciplinary approaches and protocols for treatments for behavioral addictions. The study emphasizes the multifaceted nature of internet addiction and its implications for mental health and sleep patterns.

## Introduction

The use of internet has provided several advantages and easiness in different areas of our life, although its excessive use has been a global concern. Screen time and internet usage have grown exponentially in the last few years; As of 2023, the estimated number of internet users worldwide was 5.4 billion, up from 5.3 billion in the previous year. This share represents 67 percent of global population (Petrosyan, 2024) . The Coronavirus Disease (COVID-19) pandemic contributed to this increase, once smartphones and computers became necessary tools to manage people's needs (McDowell et al., 2020).

The intense internet use, the need for more time online, the inability to control it, the functional and social impairment, anger, tension, and neglect of other activities can be described as symptoms of Internet Addiction (IA) (Young, 1998). These symptoms are related to brain alterations in areas associated with the reward system and executive functions (Cheng et al., 2023; Kuo et al., 2018; Soares et al., 2023). Abnormal decision-making behaviors, loss of attention, impulsivity, and low memorization are reported to be consequences of greater exposure to the internet in order to obtain pleasure, and allied to that, sedentary behaviors, sleeping problems and other psychological distress, such as high anxiety levels, depressive and stress symptoms have been reported (Chen et al., 2022; Khanbabaei et al., 2022; Lin et al., 2015; Marin et al., 2021; McDowell et al., 2020). These behaviors have been observed in a variety of aging groups, mainly in adolescents who are more vulnerable to them due to prefrontal formation, but young adults have also reported symptoms that might be caused by internet overuse (Andrade et al., 2020; Giedd, 2020; Lozano-Blasco et al., 2022; Marzilli et al., 2020).

A study developed in Brazil, which investigated internet addiction levels and anxiety, depression, stress, and attention deficit hyperactivity disorder (ADHD), found that those who were more addicted reported having higher averages in behavioral symptoms such as depression, stress, anxiety and lower levels of attention (Marin & Almeida, 2024). The same symptoms appear to be common with young adults and adults. Another study with 420 university students observed that the severity of IA is associated with the impact of headaches and the severity of insomnia. Internet addiction was also associated with anxiety (Rangel et al., 2022).

One of the main consequences of high screen time use and possible IA is poor sleeping quality, and it is known that establishing sleeping habits is essential for maintaining physical and mental health, which includes executive functions (Dinis & Bragança, 2018; Sen & Tai, 2023). The circadian rhythm synchronizes sleeping and the light is the primary biological clock in the suprachiasmatic nuclei, and when one is exposed to light from screens, which contain blue-enriched, short-length light, especially at night, the synchronization is affected and sleeping is impaired (Wallace-Guy et al., 2002). In a study that investigated the associations between electronic devices (ED) use before bedtime and sleep quality, it was seen that 98. 1% of the participants used at least one type of electronic device within two hours before bedtime, the smartphone, the most used ED (Pham et al., 2021).

Maladaptive sleep behavior and poor sleep tend to be associated with psychological disturbance. In a longitudinal study developed with 3,294 participants, researchers assessed Generalized Anxiety Disorder (GAD), Major Depressive Disorder (MDD) and sleeping patterns and found that poor global sleep quality functioned as a mediator of bidirectional anxiety-depression relationship (Alwhaibi & Aloola, 2023; Nguyen et al., 2022). Regarding the relationship between IA, psychological distress, and sleeping, a study conducted in Korea, with a sample of 3,212 adults, showed that poor sleep quality was associated with lifetime suicide attempts in adults with IA (Kim et al., 2017).

Apart from all psychological issues, it is known that the increased use of mobile devices before bedtime tend to be related to social media use and this phenomenon tends to be allied to the concept of fear of missing out (FOMO), which is the fear of being out of updates or outdated. (Jahrami et al., 2021; Pham et al., 2021; Przybylski et al., 2013).

Internet overuse is, therefore, a potential risk for the development of an addiction, mental health issues, sleeping problems, and social and functional impairments. Studies are necessary to investigate these factors to understand better the prevalence of this addiction and its consequences, as well as to propose interventions for prevention and treatment. The current study aims to investigate the possible associations between IA, sleep patterns, depression, anxiety, stress and FOMO levels in adolescents and young adults in Brazil. Based on previous studies, we propose two hypotheses. Hypothesis 1 is that the group with sleep problems is more addicted to the internet, and hypothesis 2 is that the internet-addicted group presents higher prevalences of mental health problems (Tereshchenko et al., 2021; Younes et al., 2016).

## Materials and methods

### Study design

An exploratory cross-sectional study was carried out from October to December 2023 in southern Brazil using a questionnaire on a survey platform (Google Forms).

### Study population

The sample was composed of* 618* adolescents and young adults of the general population aged 15–36, with a mean age of 23.34 (SD = 5.97). It was a community sample, non-probabilistic, and selected based on convenience criteria. The inclusion criteria for participation were using the internet and having the appropriate aging group.

### Instruments

#### Sociodemographic Questionnaire

A sociodemographic questionnaire was developed exclusively for this research. Data collected were related to age, exercising, and sleeping patterns (difficulties falling asleep, perceived quality of sleep, number of hours of sleep per night), sex, sexual orientation, labor information, medication use, drug use, and previously diagnosed mental health problems.

#### Internet Addiction Test (IAT)

With the aim of assessing Internet Addiction or excessive Internet use, the Internet Addiction Test (IAT) was used. The test was developed by Young (1998) and is a self-administered instrument composed of 20 questions arranged in the form of a Likert scale with points ranging from Rarely (1) to Always (5). The higher the score, the greater the severity of the dependency, which can vary from 0 to 100 points, and the results can be categorized as normal (0–30 points), light (31–49 points), moderate (50–79 points) and severe (80–100 points). Our study used the cutoff 75 (moderate to severe) of the Brazilian version of IAT scale to classify IA (Kim et al., 2012). The test used in the current study was adapted to Brazilian Portuguese by Conti et al., (2012), which showed Cronbach's alpha values very close (0.85) to those of the original study (0.54 to 0.82). In our study, the Cronbach's alpha value was 0.85.

#### Depression, Anxiety, and Stress Scale (DASS-21)

The DASS-21 scale was used to measure depression, anxiety, and stress (Henry & Crawford, 2005). It is an instrument in Likert scale format, consisting of 21 items, in which the participant selects options from 0 to 3 (from does not apply to me to it applies a lot to me). In this study, the total score of the scale with the 21 items was used.

On the scale, symptoms of anxiety and depression are grouped into three basic structures: a) presence of negative affect, depressed mood, insomnia, discomfort, and irritability; b) presence of specific symptoms of depression (anhedonia, absence of positive affect); c) specific symptoms of anxiety (somatic tension and hyperactivity) (Henry & Crawford, 2005). The test used in the current study was adapted to Brazilian Portuguese by Vignola et al. (2014), which showed Cronbach's alpha values very close (0.92 for depression, 0.90 for stress, and 0.86 for anxiety) to those of the original study (0.88 for depression, 0.82 for anxiety and 0.90 for stress). In our study, the Cronbach's alpha value was 0.95.

#### Social Media Engagement Questionnaire (SMEQ)

The SMEQ questionnaire was initially developed by Przybylski et al. (2013) aiming to understand the phenomenon of Fear of Missing Out (FOMO), which contemplates the need to stay connected to social networks. The questionnaire has five items that measure engagement on social media. The responses to the items are given using a stimulus question (how often you used a social network…), and participants must indicate the frequency of use on a response scale ranging from 0 (no day) to 7 (every day). The questionnaire was validated and adapted for the Brazilian context, presenting the same psychometric quality as the original (0.81) with an internal consistency coefficient of 0.90 (McDonald's ω) (Mariano et al., 2019). In our study, the Cronbach's alpha value was 0.76.

### Ethical procedures

The research was previously approved by the Research Ethics Committee of the Federal University of Rio Grande do Sul (CAAE: 69,692,323.6.0000.5334). All participants were informed about the nature, purposes, and possible risks of the research. They signed appropriate consent and assent forms tailored to their specific circumstances, which included detailed information on the technical and ethical aspects of the research.

### Data Analysis

The data were analyzed using Jamovi (Version 2.4; The Jamovi Project, 2023) and R (R Core Team, 2022). Descriptive statistics were computed, including mean, standard deviation, minimum, maximum, skewness, kurtosis, and frequencies. A composite score for sleep problems was calculated based on self-reported difficulty falling asleep (No/Yes) and poor sleep quality (No/Yes). Group membership was coded as 0 = Without sleep problems and 1 = With sleep problems. Levene's test was conducted to assess the homogeneity of variances before performing group comparisons and Shapiro–Wilk as well as Kolmogorov–Smirnov to assess normality. Since homogeneity was not met, Welch's t-test was used to examine differences between individuals with and without sleep problems. Independent chi-square tests were conducted to assess associations between group membership (sleep problems or not) and variables such as work status, sex, drug use, physical activity, and mental health issues. Effect sizes were reported using Cohen's d for Welch's test and Cramér's V for the chi-square test. Spearman correlations were performed to evaluate the associations among variables, including internet addiction, stress, anxiety, depression, sex, work status, drug use, and physical activity. Correlation strengths were interpreted as follows: 0 to 0.30 = small, 0.30 to 0.70 = medium, and 0.70 to 1 = large (Cohen, 1988). Diagnostic tests, including Tolerance and Variance Inflation Factor (VIF), were used to assess multicollinearity, and Cook’s distance was examined for the presence of outliers in the sample for the logistic regression model. Finally, binary logistic regression was performed to assess the variance in sleep problems explained by the predictors. The model included four continuous variables (DASS21 total scores, Internet Addiction Test [IAT] scores, Social Media Engagement Questionnaire [SMEQ] scores, and age) and three categorical variables (work status, drug use, and physical activity). The accuracy, sensitivity, and specificity of the model were analyzed. Statistical significance was set at *p* < 0.05.

## Results

The total number of participants in the study was 618, with females constituting the majority: 422 (68.3%), followed by males: 190 (30.7%), and others: 6 (1.0%). The mean age of participants was 23.34 years = 5.97). Regarding the IAT. 6.5% (*n* = 40) of the participants fell into the "normal" category (0–30 points), 44.7% (*n* = 276) were classified as having "light" internet addiction (31–49 points), 48.1% (*n* = 297) were categorized as having "moderate" internet addiction (50–79 points), and 0.8% (*n* = 5) were classified as having "severe" internet addiction (80–100 points). Regarding the variable drug use, the frequencies were: 142 (71,4%) marijuana, 14 didn't inform (7%), 5 Vape (2,5%), 33 cigarettes only (16,6%), and 5 (2,5%) used other drugs but not marijuana and cigarettes. Of the 142 subjects that used marijuana, 36 (25,4%) used other drugs such as LSD and MDMA. Table 1 shows the sociodemographic characteristics of the sample.

**Table 1:** Sociodemographic characteristics

Variables	
**Ethnicity**	
Black	14 (2.3%)
White	527 (85.3%)
Brown	71 (11.5%)
Indigenous	1 (0.2%)
Asian	5 (0.8%)
**Sexual Orientation**	
Heterosexual	490 (79.3%)
Homosexual	35 (5.7%)
Bisexual	84 (13.6%)
Others	9 (1.5%)
**Work status**	
Employed	520 (84.1%)
Unemployed	98 (15.9%)
**Physical Activity**	
Physically Active	432 (69.9%)
Not Physically Active	186 (30.1%)
**Sleep Difficulties**	
Yes	262 (42.4%)
No	356 (57.6%)
**Drug Use**	
Yes	199 (32.2%)
No	419 (67.8%)
**Mental Health Problems**	
Yes	208 (33.7%)
No	410 (66.3%)

IAT scores ranged from 21 to 91 (M = 49.37, SD = 12.74), DASS21 scores ranged from 0 to 63 (M = 20.58, SD = 13.85), SMEQ scores ranged from 0 to 35 (M = 21.82, SD = 8.63). The results from Welch's test between groups with and without self-reported sleep problems revealed significant differences across most study variables. Individuals with sleep problems were slightly older, with a mean age of 23.8 years compared to 22.8 years in those without sleep issues, with a small effect size (d = 0.173). Notably, those with sleep problems reported fewer sleep hours, averaging 6.28 h per night compared to 6.86 h in the group without sleep problems, with a large effect size (d = 0.629), highlighting a substantial difference in sleep duration.

Regarding our mental health measures, the group with sleep problems showed significantly higher scores on the Internet Addiction Test (IAT), with a medium effect size (d = **-**0.504). Similarly, significant differences were observed in the Depression, Anxiety, and Stress Scale (DASS-21) scores; the sleep problem group had much higher levels of stress, anxiety, and depression, all with large effect sizes (d = **-**0.726, d = **-**0.712, and d = **-**0.724, respectively). Social media engagement (SMEQ) was also slightly higher in the sleep problem group, although the effect size was small (d = **-**0.216).

Significant associations were found between sleep problems and work status, physical activity, and mental health problems in terms of categorical variables. Specifically, a higher proportion of individuals with sleep problems reported being unemployed and having mental health issues. Conversely, physical activity was less common in the sleep problem group. There were no significant differences between the groups in terms of drug use or sex. Table 2 compares the demographics and test results between two groups divided according to their self-reported sleep problems.

**Table 2:** Group comparisons according to self-reported sleep problems

Variables	Group without sleep problems (*n* = 356)	Group with sleep problems (*n* = 262)	*p*	Effect Size
Age	22.8 ± 5.84	23.8 ± 6.05	*0.033	0.173
Sleep Hours	6.86 ± 0.70	6.28 ± 1.11	*0.001	0.629
IAT	46.7 ± 12.04	53.0 ± 12.81	*0.001	-0.504
DASS21	16.1 ± 11.86	26.6 ± 14.12	*0.001	-0.802
Stress	6.54 ± 4.42	9.94 ± 4.92	*0.001	-0.726
Anxiety	4.09 ± 3.99	7.32 ± 5.02	*0.001	-0.712
Depression	5.50 ± 4.74	9.33 ± 5.76	*0.001	-0.724
SMEQ	21.0 ± 8.73	22.9 ± 8.39	*0.008	-0.216
Work status, No/Yes	41/315	57/205	†0.001	.139
Physical Activity, No/Yes	93/263	93/169	†0.012	.101
Drug Use, No/Yes	246/110	173/89	†0.419	.032
Mental Health Problems, No/Yes	261/95	149/113	†0.001	.172
Sex, Male/Female	112/242	78/180	†0.710	.015

*IAT* Internet Addiction Test, *DASS-21* Depression, Anxiety and Stress Scale, *SMEQ* Social Media Engagement Questionnaire

^*^The comparison was performed using independent samples of Welch's test

^†^The comparison was performed using a chi-squared test

Regarding the Spearman correlations, Age showed weak but significant negative correlations with DASS-21, stress, and anxiety, suggesting that younger individuals may experience higher levels of these psychological symptoms. Age also had a positive correlation with work status and drug use, indicating that older individuals were more likely to be employed and engage in substance use. IAT scores were positively correlated with DASS-21, stress, anxiety, depression, and social media engagement. IAT had a negative correlation with work status, indicating that those with higher internet addiction levels were more likely to be unemployed.

DASS-21 had moderate positive correlations with sex, with women reporting higher distress levels and a negative correlation with physical activity, indicating that more physically active individuals had lower psychological distress. Sex negatively correlated with physical activity, suggesting that females were less likely to engage in physical activity. Work status was negatively correlated with DASS-21, stress, anxiety, and depression, indicating that employed individuals reported lower levels of psychological distress. Additionally, work status was positively correlated with drug use, suggesting that those who were employed were more likely to use drugs. Table 3 presents the Spearman correlations between study variables.

**Table 3:** Correlation between study variables

	**Age**	**IAT**	**DASS21**	**Stress**	**Anxiety**	**Depression**	**Gender**	_**Work status**_	**Drug Use**	**Physical Activity**
Age	-									
IAT	-.052	-								
DASS21	-.114**	.415***	-							
Stress	-.115**	.367***	.912***							
Anxiety	-.096*	.307***	.899***	.800***	—					
Depression	-.078	.434***	.895***	.708***	.695***	—				
Gender	.173***	.076	.301***	.317***	.326***	.215***	—			
Work status	.262***	-.132**	-.150***	-.128***	-.110**	-.161***	.015	-		
Drug Use	.189***	.100*	.116**	.092*	.096*	.129***	.034	.110**	**-**	
Physical Activity	_.006_	-.058	-.179***	-.141***	-.171***	-.200***	-.153***	.072	.037	-
SMEQ	-.029	.340***	.214***	.193***	.158***	.219***	.052	-.008	.123**	-.031

*IAT* Internet Addiction Test, *DASS-21* Depression, Anxiety and Stress Scale, *SMEQ* Social Media Engagement Questionnaire

^*^p < .05, ** *p* < .01, *** *p* < .001

The binary logistic regression model predicting sleep problems was significant (χ^2^ = 106, df = 7, *p* < 0.001), with a Nagelkerke R^2^ of 0.213, suggesting that approximately 21.3% of the variance in sleep problems could be explained by the model. The model correctly classified 68.4% of cases, with a sensitivity of 51.9% and a specificity of 80.6%, showing a moderate ability to predict sleep problems. The area under the curve (AUC) was 0.731, indicating a fair level of discriminative ability. This model did not consider multicollinearity, as the Variance Inflation Factor (VIF) values ranged from 1.02 to 1.21, with corresponding tolerance values between 0.82 and 0.97.

In terms of individual predictors, both the Internet Addiction Test (IAT) and the Depression, Anxiety, and Stress Scale (DASS-21) were significant predictors of sleep problems. Specifically, higher IAT scores were associated with increased odds of reporting sleep problems (*p* = 0.016, OR = 1.020), and higher DASS-21 scores were also linked to higher odds of sleep issues (*p* < 0.001, OR = 1.055). It suggests that individuals with higher internet addiction and greater psychological distress are more likely to experience sleep problems. Other predictors, including age, social media engagement, physical activity, drug use, and work status, did not significantly predict sleep problems in this model. Table 4 presents all study variables’ parameter estimates.

**Table 4:** Binary logistic regression predicting Sleep Problems

						**95% Confidence Interval**	
**Predictor**	**Estimate**	**SE**	Z	**p**	**Odds ratio**	**Lower**	**Upper**
**Intercept**	-1.684	.582	-2.891	.004	.185	.059	.581
**Age**	-.007	.015	-.452	.651	.993	.962	1.024
**IAT**	.0194	.008	2.408	.016	1.020	1.004	1.036
**DASS21**	.0540	.007	6.897	.001	1.055	1.039	1.072
**SMEQ**	-.0008	.011	-.078	.938	.999	.977	1.021
**Work:**							
Yes – No	-.459	.254	-1.807	.071	.632	. 384	1.040
**Drug Use:**							
Yes – No	-.035	.197	-.180	.857	.965	.655	1.421
**Physical Activity:**							
Yes – No	-.181	.194	-.928	.353	.834	.569	1.223

Estimates represent the log odds of "Sleep Difficulties = Yes" vs. "Sleep Difficulties = No."

*IAT* Internet Addiction Test, *DASS-21* Depression, Anxiety and Stress Scale, *SMEQ* Social Media Engagement Questionnaire

## Discussion

The present study explored the associations between IA, sleep patterns, and psychological distress, including depression, anxiety, and stress, in adolescents and young adults in Brazil. The findings underscore the importance of understanding this population's complex interplay between excessive internet use, sleep quality, and mental health.

The prevalence of IA in the studied sample is 48.1% (*n* = 297) were categorized as having moderate internet addiction (50–79 points), and 0.8% (*n* = 5) were classified as having severe internet addiction (80–100 points). Our result is consistent with previous international and national research (Cadena & Taramuel, 2022; Kim et al., 2012; Marin et al., 2024). In one of them, which aimed to investigate the value of IAT for internet addiction, it was observed that 42% of the subjects had significant IA (Kim et al., 2012). That aligns with the global concern about the escalating trend in internet usage, especially exacerbated during the COVID-19 pandemic (Singla et al., 2023). The substantial increase in screen time, as evidenced by the Internet Growth Statistics (n.d.), indicates a potential risk for developing IA.

Consistent with previous research, our study revealed a significant relationship between IA and poor sleep quality (Hammad et al., 2024). The negative impact of excessive screen time on circadian rhythm and the use of electronic devices before bedtime has been previously established (Pham et al., 2021). The association between IA and sleep problems is concerning, as disrupted sleep patterns can contribute to various psychological issues, including anxiety and depression. 

The correlations presented in Table 3 highlight the interconnectedness of variables. IA was positively correlated with depression, anxiety, and stress, emphasizing the multifaceted nature of internet addiction and its potential implications for mental health. Additionally, the negative correlation between age and psychological distress suggests that younger participants may be more susceptible to anxiety and stress, aligning with existing literature (Andrade et al., 2020; Giedd, 2020; Nikolic et al., 2023).

The logistic regression analysis provided valuable insights into the predictors of sleep problems. Notably, both IA and overall psychological distress, as measured by the DASS-21, were associated with an increased likelihood of experiencing sleep problems. That aligns with previous research indicating that poor sleep quality can mediate the bidirectional relationship between anxiety and depression (Nguyen et al., 2022). The inclusion of IA as a predictor further highlights its role as a significant factor influencing sleep patterns. In a study which aimed to investigate the associations between IA, depression, insomnia and self-esteem in students, the results corresponded to ours, showing that IA is associated with depression, insomnia and low self-esteem (Cadena & Taramuel, 2022).

Work status emerged as another noteworthy predictor since IAT had a negative correlation with work status, indicating that those with higher internet addiction levels were more likely to be unemployed. This phenomenon was also mentioned in other studies (Rumpf et al., 2013; Müller et al., 2013). During the COVID-19 pandemic, several people lost their jobs, and the effects were related to mental disorders, such as anxiety and being into debt, showed to have a positive and significant effect on insomnia (Zamanzadeh et al., 2023). As anxiety levels are high, the tendency to overuse the internet is also high, being considered as a coping behavior (Augner et al., 2023). The potential bidirectional relationship between unemployment, sleep problems, and internet addiction warrant further investigation.

In our study, we also found some associations between sleeping problems and social media use, including FOMO (Przybylski et al., 2013). Some other studies also found this relationship (Levenson et al., 2016; Pham et al., 2021; Silva et al., 2022). In one of them, developed with college students, it was seen that approximately half of them had poor sleep quality, and 98.1% used at least one type of electronic device within two hours before bedtime for recreation, social networking, or studying without adjusting screen light (Pham et al., 2021).

Furthermore, our results suggested that 185 participants reported in the sociodemographic questionnaire that they use drugs, such as marijuana (71,4%), and this result implies that those who use drugs also report more psychological distress symptoms and more sleep problems. Our finding extends another study, which showed that daily marijuana users endorsed more sleep disturbance than non-daily users, but, on the other hand, our study contradicts the correlation with psychological distress, which means that further research should investigate this phenomenon (Conroy et al., 2016).

Finally, it is relevant to mention that excessive internet use is considered a risk factor for the development of sleep deprivation, psychopathology and executive function impairments, and might cause losses in social and functional areas in the short and long term. Our study further elucidates the role of internet addiction in sleep problems and psychopathology (Chen et al., 2022; Khanbabaei et al., 2022; Lin et al., 2015; Marin et al., 2021; McDowell et al., 2020). On the other hand, we observed that the participants who practice physical activity experience less psychological distress and sleep better. The same was observed in other studies (Schuch & Vancampfort, 2021).

While the study provides valuable insights, some limitations should be acknowledged. The cross-sectional design prevents the establishment of causal relationships, and self-report measures may introduce biases related to socially desirable responding (SDR), that is the people's tendency to present a favorable image of themselves on questionnaires (Mortel., 2008). Future research should consider longitudinal approaches and objective sleep assessments to enhance the robustness of findings. The heterogeneity in terms of an extensive age range of the participants is also seen as a limitation of the study, once there are differences in terms of the development characteristics. Another limitation of the study is the external generalization of the study since the sample is comprised mainly of young people. Thus, the sample does not represent the entire Brazilian population, as the participants are mostly made up of black and brown adolescents and young adults.

In conclusion, this study contributes to the growing body of literature addressing the intricate relationship between internet addiction, sleep patterns, and psychological distress in adolescents and young adults. The findings underscore the need for targeted interventions and preventive measures to mitigate the adverse effects of excessive internet use on mental health and sleep quality in this vulnerable population. Also, we support the importance of addressing IA in younger populations through educational programs, mental health support, and regulation of screen time. Public health strategies should include IA and its consequences in health programs with multidisciplinary approaches (psychotherapists, teachers, physicians, professors). Protocols for treatments for behavioral addictions should be developed. Due to its importance, it is suggested that this topic be included in schools, universities, groups, and general society through lectures, information booklets, and media dissemination (Chun et al., 2017; Young, 2007).

## Conclusion

The internet has been a useful and necessary tool in our lives, but the high frequency and intensity of use have been a global concern. The consequences of internet overuse might lead to Internet addiction and several other consequences, such as sleeping problems and other psychological distress. This study identified relationships among internet addiction, stress, depression, anxiety, sleeping difficulties, FOMO, work and drug use in adolescents and young adults. The variables stress, anxiety and depression were all correlated to IA and sleeping difficulties, sustaining the negative impact of internet overuse and corroborating other studies in the field. Not having a job is seen to be an important risk factor for sleeping problems and IA, but further research in the area should be conducted. Struggling to sleep is related to higher social media use and FOMO. There is a need for targeted interventions and preventive measures to mitigate the adverse effects of excessive internet use on mental health and sleep quality. Public health strategies should include IA and its consequences in health programs with multidisciplinary approaches, and Protocols for treatments for behavioral addictions should be developed. Further research should be conducted not only in the assessment of IA and its consequences, but also in prevention and treatment strategies.

## Data Availability

The datasets generated and/or analyzed in the current study are available from the corresponding author upon reasonable request.

## Abbreviations

COVID-19: Corona Virus Disease
IA: Internet Addiction
GAD: Generalized Anxiety Disorder
MDD: Major Depressive Disorder
ADHD: Attention Deficit Hyperactivity Disorder
ED: Electronic Devices
FOMO: Fear of Missing Out
IAT: Internet Addiction Test
SD: Standard Deviation
DASS-21: Depression, Anxiety and Stress Scale
SMEQ: Social Media Engagement Questionnaire
CMD: Common mental disorders
SDR: Social desirability responding
X2: Chi Square Test
